# Application of Measurement Sensors and Navigation Devices in Experimental Research of the Computer System for the Control of an Unmanned Ship Model

**DOI:** 10.3390/s21041312

**Published:** 2021-02-12

**Authors:** Tadeusz Szelangiewicz, Katarzyna Żelazny, Andrzej Antosik, Maciej Szelangiewicz

**Affiliations:** 1 Faculty of Navigation, Maritime University of Szczecin, Wały Chrobrego 1-2, 70-500 Szczecin, Poland; k.zelazny@am.szczecin.pl; 2 Company Smart-Electronics, 72-002 Dołuje, Poland; andrzej.antosik@smart-electronics.eu; 3 Company Conmar, 71-075 Szczecin, Poland; maciej.szelangiewicz@icloud.com

**Keywords:** autonomous unmanned vessel (MASS), computer control system, measuring sensors and navigation devices, model tests of unmanned vessel in open water, recording of movement parameters and images from lidars and cameras

## Abstract

Unmanned autonomous transport vessels (MASS) are the future of maritime transport. The most important task in the design and construction of unmanned ships is to develop algorithms and a computer program for autonomous control. In order for such a computer program to properly control the ship (realizing various functions), the ship must be equipped with a computer system as well as measurement sensors and navigation devices, from which the recorded parameters are processed and used for autonomous control of the ship. Within the framework of conducted research on autonomous ships, an experimental model of an unmanned ship was built. This model was equipped with a propulsion system not commonly used on transport vessels (two azimuth stern thrusters and two bow tunnel thrusters), but providing excellent propulsion and steering characteristics. A complete computer system with the necessary measuring sensors and navigation devices has also been installed in the model of the ship, which enables it to perform all functions during autonomous control. The objective of the current research was to design and build a prototype computer system with the necessary measurement sensors and navigation devices with which to autonomously control the unmanned ship model. The designed computer system is expected to be optimal for planned tasks during control software tests. Tests carried out on open waters confirmed the correctness of the operation of the computer system and the entire measurement and navigation equipment of the built model of the unmanned transport vessel.

## 1. Introduction

In maritime transport, since the beginning of the 21st century, new technological solutions have been designed and researched to lead to:

- Improvements and further increases in the safety of maritime transport: the analyses of maritime accidents and catastrophes carried out indicate that their cause is not a failure of the ship’s equipment, but only human error (depending on the type—collision, grounding, fire—man is responsible for from 80–96% of all accidents, [1,2,3];
- Reduction in ship operation costs: about 20–30% of these costs is the maintenance of the crew and shipowners’ services dealing with the crew;
- Environmental protection: although maritime transport accounts for about 3% of the world’s CO_2_ emissions to the atmosphere, new ship designs are expected to have completely green propulsion systems;
- Improvements in other indicators affecting the economic side of maritime transport: new hull design with less weight will result in better use of ship displacement;
- More accurate running of ships on the shipping lines, which can lead to a reduction in energy expenditure, reduced voyage time, or improved punctuality of entry into the port of unloading.

Such a new solution in maritime transport, which will meet higher expectations, is unmanned autonomous vessels. The most important task during the design and construction of autonomous ships is to develop computer control systems. Such a system has to be equipped with special software that allows the determination of the route of the voyage and the realization of various tasks (maneuvers) during the autonomous control, as well as starting of the remote control during e.g., change in the autonomy level because of various emerging threats or difficult tasks during the operation of the ship, e.g., voyage through areas with a high intensity of ship traffic or entry into ports.

In order for the software installed in the computer system to be able to perform various tasks during autonomous control, the ship must be equipped with various measuring and navigation devices. This paper will present an experimental model of an autonomous transport ship, equipped with a computer control system and the necessary measurement and navigation equipment. A prototype of the measurement and navigation system will be presented, as well as research and tests of the use of measurement sensors and navigation devices for the autonomous control of an unmanned ship model.

## 2. Status of Research on Autonomous Control Systems for Unmanned Ships

The first research work on the possibility of building unmanned autonomous transport vessels began in the early 21st century.

In 2013, Rolls-Royce Marine began working on the design of an autonomous ship. The Advanced Autonomous Waterborne Applications Initiative (AAWA) project was launched to develop the design and technical solutions (navigation, safety, monitoring, collection, and processing systems) for building autonomous vessels. The AAWA project has also developed a virtual center for controlling the fleet of unmanned vessels [4].

Currently, research and experimental work on MASS’s are being carried out worldwide in two directions:

- Construction and testing of remote and then autonomous control systems on existing crew ships (usually small car-passenger ferries operating at short distances);
- Design, construction and experimental research of unmanned transport vessels in the first stage, these are physical models of ships with a length of several meters and then the construction of target unmanned vessels.

Examples of conducted tests of autonomous control systems installed on unmanned ships are listed below:

- Tests of autonomous control on Falco’s Finnish car ferry—December 2018 [5]: the ferry performs autonomous maneuvers, is supervised by an operator from the land-based center in Turku. No information about the applied measurement and navigation devices, algorithms, and control programs is available.
- Tests of autonomous control of the ferry Suomenlinna II—Finland, December 2018 [6]: tests monitored from the land-based center in Helsinki. No information about the applied measuring and navigation devices, algorithms, and control programs.
- Autonomous control tests on the supply vessel SeaZip 3, the Netherlands, in the North Sea—March 2019 [7]: no information about the applied measuring and navigation devices, algorithms and control systems.

**Figure 1:**
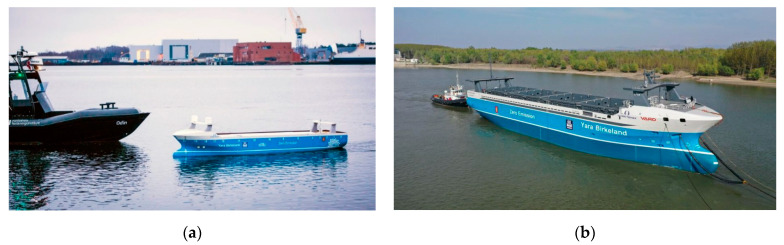
Model of the ship Yara Birkeland for research and testing (**a**) [9], and the autonomous container ship Yara Birkeland launched in February 2020 (**b**) [10] (120 TEU, L = 79.5 m, B = 14.8 m).

Examples of projects and studies for new unmanned autonomous vessels are listed:

- The Re-Volt—remote container ship with electric drive (Norway) [8]: designed from 2017 by DNV GL, it is to carry 100 TEU; there is no information about the ship’s construction and propulsion and no information about the applied, for autonomous control, measuring and navigation devices, and about the effects of experience from the realized project.
- Ship Yara Birkeland—designed since 2017 unmanned container ship (Norway) [9]. Figure 1 shows model tests in 2018.

Apart from the pictures of the model and the launched ship (Figure 1), there is no information about the results of the model tests, about the applied measuring and navigation devices, about the construction of the built ship, and about the algorithms and systems of remote and autonomous control of the ship.

There are also other projects, such as the ShippingLab Project (Denmark), SeaShuttle Project (Norway and the Netherlands), Autoship Project (United Kingdom R-R and Norway Kongsberg), but also in these cases, there is no practical information available.

The information about the implemented unmanned ship transport projects is placed on the Internet in the form of notes and not in scientific publications. Apart from modest information about conducted trials, they do not contain any important information about specialist equipment, construction of a control system, and about the algorithms and computer control programs.

In addition to online information on research or tests of autonomous control systems for unmanned transport vessels, a few articles have been published on other unmanned waterborne objects, e.g., small, unmanned hydrodrones for measurement or research. The publication [11] presents a concept and electronic system for planning bathymetric measurements in shallow waters using an autonomous unmanned MASS. The article lacks data concerning the vehicle structure and control algorithms. The article [12], consisting of two parts, presents the classification of floating autonomous objects, a proposal of autonomy levels, and an extensive review of prototypes of autonomous units without information about the design, construction, and results of autonomous control tests.

The most information on the structure and equipment of hydrodrones for hydrographic research is presented in [13]. The article presents a functional diagram of the hydrodrone with a description of the possibilities of realizing different levels of autonomy, hardware architecture with a list of measuring and navigation devices used during operation at selected autonomy levels, and software architecture. The construction of the hydrodrones with the measurement and navigation sensors installed was not included, nor were the results of tests and studies conducted in the open water.

Publications [14,15,16,17] present the results of tests in the basin model carried out on remote-controlled models equipped with navigation devices and a computer system for autonomous execution of some maneuvers. Publications [14] and [15] examine the possibility of cruising the model ship “Essp Osaka” on a given trajectory and performing circulation tests. The publication [16] presents a control and measurement system for real time maneuvering of the transport ship model. The publication [17] presents a computer system for research and evaluation of autonomous maneuvers to avoid collision at sea. In these publications, a model of a transport vessel with classic propulsion was examined: one propeller and one finned rudder placed behind the propeller. In addition to these publications, there are also articles on dynamic systems of vessel positioning and movement along a given trajectory. These publications include control algorithms, a description of the control and measurement system installed, and the results of tests carried out. The information from these publications can also be useful for the design, construction, and testing of unmanned transport vessels with autonomous control.

Subsequent publications [18,19,20,21] presented the results of computer simulation studies and experiments of autonomous ship models. The studies presented there concerned the detection of obstacles at sea by means of radar, lidar, sonar, or cameras and the determination of a safe navigation route. The configuration of the computer systems used, the technical parameters of these systems, and the measurement and navigation equipment were not included; neither are the methods of measurement data processing nor their registration and transmission to land-based centers monitoring the movement of the autonomous vessels presented.

## 3. Purpose, Scope, and Test Method

The analysis of the available literature shows that although research and tests of autonomous control systems for floating objects are being carried out, there is very little relevant information given in these publications useful for practical use in the design and construction of autonomous control systems for unmanned transport vessels.

The requirements and functions to be performed by an unmanned, autonomously operated transport ship are defined mainly in IMO (International Maritime Organization) regulations and in regulations of Classification Societies. It follows from these regulations that the ship must be equipped with a computerized control system enabling the change of autonomy level—switching to remote control by the operator (navigator) from the land-based center. In addition, the equipment of the ship with measuring sensors and navigation devices must provide full navigational information and diagnostic information about the operation of all devices on the ship (mainly propulsion). An example of the complexity of navigation equipment is the proposal of an integrated navigation bridge on an autonomous ship presented in the publication [22]. The anticipated navigation and measurement equipment on an actual ship are very sophisticated; there are various devices and sensors measuring the same parameters (there must be an appropriate level of redundancy required by the relevant regulations).

Since it is very expensive to build an unmanned transport vessel and to carry out tests on the autonomous controlling system, such tests are carried out on physical models of unmanned vessels.

The analyzed scientific publications lack information on the configuration of the computer system with measurement and navigation equipment. There is also no information on the technical parameters of the measurement and navigation equipment and the on-board computer, in particular its computing power necessary for autonomous control of the unmanned ship model.

The aim of the conducted research on unmanned transport vessels is the design and construction of a prototype model of the ship together with a computer system and the development of software for autonomous control. The autonomous control system must perform the following functions:

- Automatic mooring/unmooring to the quay;
- Selection and optimization of the shipping route from one port to another;
- Maritime detection of fixed and mobile objects (other vessels);
- Possibility to switch to remote control (change of autonomy level);
- Performing anti-collision maneuvers;
- Electronic communication between ships;
- Other functions (after the formulation of rules for autonomous vessels).

Checking if the developed computer program will correctly perform the listed functions will be carried out by the experimental method using a model of an unmanned vessel and by the simulation method (computer simulation). The whole research task has been divided into two stages. The first stage of the research is the design and construction of an experimental model of an unmanned vessel and its equipment with: a propulsion system, computer control system, and all those necessary for autonomous control sensors and navigation devices. The second stage is the development of algorithms and a computer program for autonomous control.

The objective of stage I is to develop and build a computer control system for a model of an unmanned vessel under the following assumptions:

- The propulsion system of the model of the ship must be ecological and emission-free with smooth regulation of control parameters;
- Computer system parameters (computing power, memory capacity, speed of processing measurement data, their recording in the memory, and transmission to the Ground Control Station (GCS) must be adjusted to the volume (number) and frequency of recorded parameters;
- The set of measurement sensors and navigation devices must be able to perform all of the above functions;
- Technical and operational parameters of the computer system components (e.g., dimensions, range, measurement accuracy, etc.) must be appropriate for the ship model (dimensions, speed of movement of the ship model—Table 1);
- The cost of building the system should be as low as possible.

The scope of the study for the implementation of stage I performed by the experimental method in open water is to check:

- Correct operation of the computer system, measurement and navigation sensors, and registration of all parameters;
- Correctness of processing and sending received signals from these sensors to the computer control system;
- Computing capacity of a computer made in industrial technology receiving and processing the recorded and measured parameters from measurement sensors and navigation devices;
- The correctness of sending the recorded parameters from the measurement and navigation sensors in real time to GCS;
- Correct detection of obstacles set on the water and performing anti-collision maneuvers;
- The correctness of switching to manual or remote control with GCS or when changing the autonomy level.

## 4. Experimental Model of an Unmanned Transport Vessel Controlled Autonomously

Source data analyses suggest that the first unmanned transport autonomous vessels will be small container ships operating in internal waters or dedicated waters [10], equipped with environmentally friendly electric propulsion systems.

**Figure 2:**
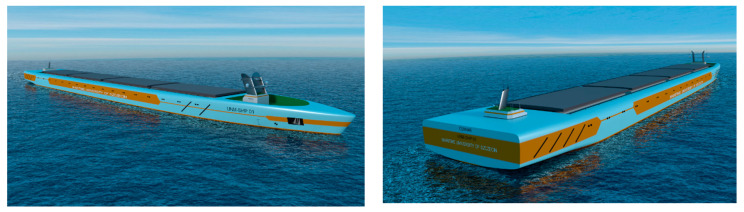
Project visualization of an unmanned container ship (bulk carrier), controlled autonomously.

**Figure 3:**
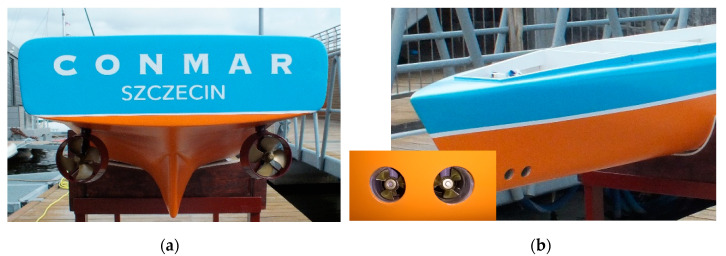
Unmanned vessel model propulsion system: (**a**) azimuthal stern thrusters, (**b**) bow tunnel thrusters.

Based on these projections, in 2018, the project of an unmanned container ship (bulk carrier, Table 1), autonomously controlled, was made (Figure 2). The model of the unmanned ship in scale 1:25 (Figure 3) was built for testing of the autonomous control system (Table 1).

The experimental model of an unmanned ship has been equipped with an ecological, electric propulsion system of the same design as the actual ship. The propulsion system was designed so that the actual ship (and its model) would have excellent propulsion and maneuvering characteristics and could autonomously (or remotely) perform all possible maneuvers. The propulsion system consists of (Figure 3):

- Two stern azimuthal thrusters;
- Two bow tunnel thrusters.

This type of propulsion of the ship (or model) allows for very accurate and safe execution of all necessary maneuvers during autonomous control without human intervention.

The hydrodynamic characteristics of the model propellers were tested experimentally in the model basin (description of the research and measured characteristics are included in [23]).

Planned research and tests of the autonomous control system (automatic mooring, selection and optimization of the navigation route, obstacle detection at sea and anti-collision, and other maneuvers) require a land-based system for controlling and monitoring the movement of the ship model and a computerized control system installed in the ship model.

The computer control system installed in the model consists of:

- PC-class computer;
- I/O cards (analog–digital and digital–digital);
- Individual controllers for each drive motor;
- RC receiver (remote control in emergency situations or change of autonomy level);
- Internal Ethernet network;
- External antennas for communication on different frequencies;
- A set of measuring sensors and navigation devices adequate for the model of an unmanned transport vessel and planned tests of autonomous control.

The control computer is an industrial-grade PC mini equipped with a passive cooling system, SSD drive, and Intel i7 processor (Advantech, Warszawa, Poland). The parameters of the control computer was sufficient for:

- Processing data from all sensors;
- Performing calculations resulting from ship model control algorithms;
- Machine learning using artificial intelligence.

All these computing tasks must be performed in real time.

The computer works with an I/O module whose purpose is:

- Conversion of data sent by the computer into motor control signals—PWM;
- Sensor readings for measuring the power consumed by motors;
- Reading the system supply voltage;
- Reading data from the ship’s emergency manual control system;
- Reading data from the direct control receiver.

The I/O module uses an ARM^®^ 32-bit Cortex^®^-M3 single-chip microcontroller running at 72 MHz.

When controlling the drive motors, the value and direction of rotation is determined by applying a PWM signal from the I/O module at a frequency of 400 Hz and a pulse width of 1000–2000 µs (neutral, motor stop 1500 µs). The PWM signal is generated in the I/O module by an 8-channel PWM generator with a resolution of 12 bits controlled by a bidirectional I2C digital bus controlled by the microcontroller.

The use of a separate I/O module with its own software instead of directly controlling the motors and reading their parameters via a PC was dictated by safety and functionality considerations:

- The I/O module has a Fail Safe mode—in case of suspension of the PC software during testing new algorithms, it allows one to take over manual control of the ship model;
- Independent power system monitoring and response to battery charge status;
- Prevents accidental startup of drive systems during software testing;
- Provides a long-range emergency communication channel;
- It is possible to modify the I/O module software to implement a simple software controlling system, which, in the event of a Fail Safe condition, will allow the ship model to return to a preset position.

The I/O module uses several Fail Safe systems for the following events:

- Suspension or lack of communication between the PC and I/O module: the software sends cyclically, in addition to control and read commands, a data frame called heart beat—a frame of a few bytes sent at a software-controlled frequency of 1–0.1 Hz as a request–response. If the frame does not arrive in the expected time, the I/O module stops the thrusters and switches the control mode to manual.
- I/O Module Software Suspend: Software Watchdog 500 ms (can be reduced if necessary).
- Loss of the manual control signal (priority—the system provides for manual control of the ship model in every situation): then, the thrusters are stopped (worst case scenario) and the ship model has to be recovered by itself. It is possible to extend the I/O module by a function for automatic ship model return.
- Supply level too low: A two-stage action is provided. When the first level is exceeded, the I/O module will not allow a high-power task. When the second level is exceeded, the thrusters are stopped, but the system can be switched to manual mode and the thrusters can be forced to work manually until the batteries are completely discharged—this is an extreme emergency situation and destructive for the batteries, but in some situations, it must be possible to move the model ship away from a dangerous place, e.g., to avoid a collision. All values are configured in the I/O module. The operator can view these data in the GCS and is informed in advance when the critical values are approached.

The system uses a 115,200 bit/s serial link for communication between the PC and the I/O module. Communication based on commands issued in ASCII format has been developed for the system. The time delay for the I/O module to respond to a query from a PC is between 50 and 100 ms. To speed up communication, it is possible to change communication from ASCII to binary format.

Due to the variety of data exchange systems between subsystems, the computer has a driver software, processing data from sensors and I/O module to a common JSON protocol, with which the software controlling the ship model communicates. In addition, the driver has a module to log data for further analysis.

The communication system with the Ground Control System (GCS) uses a broadband network operating in the 2.4 and 5 GHz bands, and the 433 or 868 MHz Long Range System (LRS) low-bandwidth band for emergency communications. Two omnidirectional antennas with 3.5 dBi gain for the 2.4 GHz band and 5 dBi gain for the 5 GHz band and a dual-band directional panel antenna with 9 dBi gain for the 2.4 GHz band and 11 dBi gain for the 5 GHz band in GCS were used for MASS communications. In addition to broadband communications, basic exchange of telemetry and control data with the MASS is conducted at all times via a narrowband 433 or 868 MHz LRS-type link, which allows for control of the MASS when other communications systems fail.

By using a PC-class computer and internal LAN MASS, it is possible to install any other communication systems. 

The GCS system is based on a built-in industrial PC-class computer with an LCD monitor. Due to the extensive communication systems and safety systems (Fail Safe), the MASS manual control system is based on a separate proportional control controller, a separate LRS narrowband communication module, and a dual 2.4 and 5 GHz broadband communication system, as well as having a built-in LTE modem.

## 5. Measuring Sensors and Navigation Devices Used in the Autonomous Control System

The developed computer system of autonomous control, during the ship’s model task (e.g., cruise on a given route and performing an anti-collision maneuver), must receive the necessary information concerning not only the vessel’s movement parameters (position, speed, course), but also information or warnings about an emerging fixed or moving obstacle (another floating vessel).

**Figure 4:**
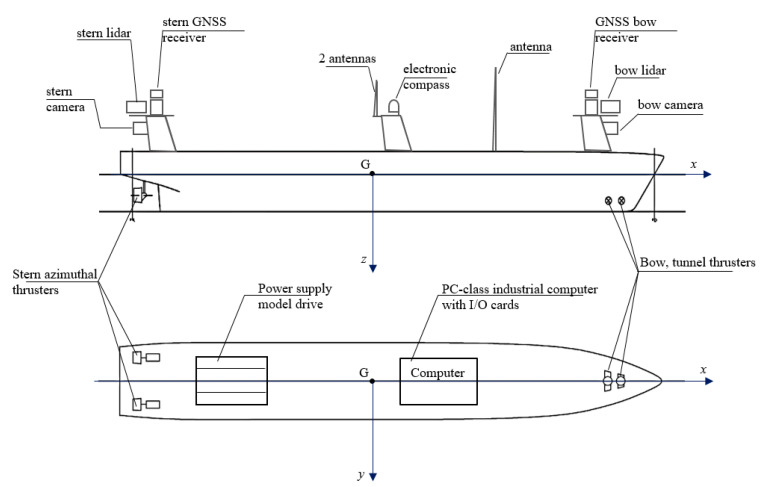
Computer system and navigation and measurement devices in the transport model of an autonomous ship.

**Figure 5:**
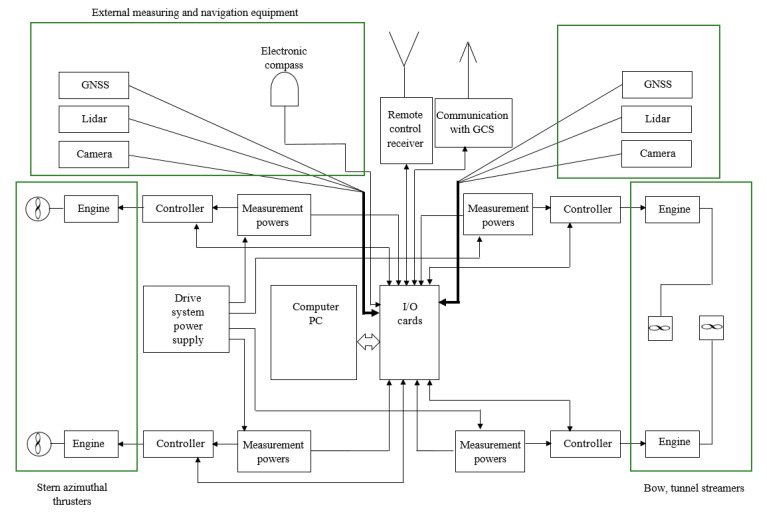
Block diagram of the unmanned ship control system, with navigational measurement devices.

The model of the unmanned vessel has been equipped with the following measurement and navigation devices, connected to the I/O cards of the computer system (Figure 4 and Figure 5):

- Lidars working in the 360° range (bow and stern);
- Global Navigation Satellite Systems (GNSS) receivers (bow and stern);
- Electronic compass;
- HD cameras (bow and stern).

The block diagram of the computerized control system of the unmanned vessel transport model is shown in Figure 5.

The parameters of the measuring and navigation sensors installed in the ship’s model have been selected so that the measured values are sufficiently accurate for the model with specified dimensions and speed of sailing (Table 1) and for the planned tests on the water during calm weather (no undulations and high wind speed).

Laser lidars for measuring the distance and angular position of the ship from an obstacle in the 2D plane in the range of 360˚, measurement frequency from 5 to 15 Hz, time of a single measurement 0.25 ms, range of the rangefinder from 0.15 to 12 m, and data transmission—115.2 kbit/s via USB port.

Cameras: for observation of the environment in the bow of the model and behind the stern, recording and transmitting HD video using IP protocol, and data transmission is via Ethernet, using an encoding system (H264 or H265 codec).

GNSS receivers: for measuring the position and speed of the model ship, data transmission 9.6 kbit/s, via NMEA protocol, USB port.

Electronic compass: for ship course measurement, a three-axis compass with electronic inclination compensation, data transmission 4.8 kbit/s, NMEA protocol, USB port.

Parameters (images) from measurement and navigation devices are recorded and transmitted in real time to the I/O module and to GCS. To integrate all elements of the computer system, an Ethernet switch was used.

## 6. Results of Tests of the Computer System and Measurement and Navigation Devices

**Figure 6:**
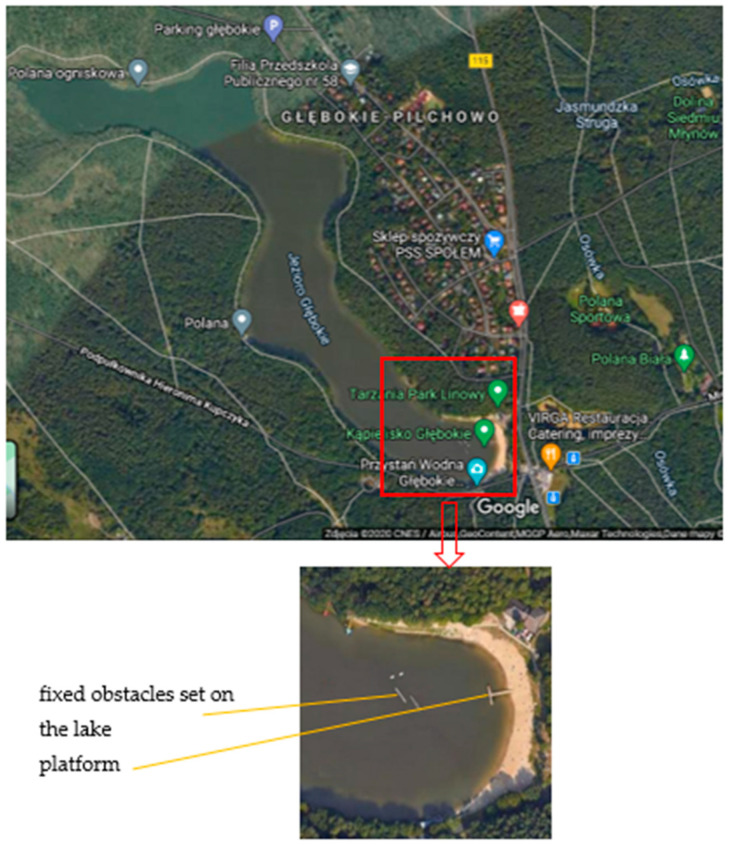
“Głębokie” Lake, which has been tested on for the system of autonomous control of an unmanned ship model [24].

Experimental research on the model of an unmanned ship equipped with a computer system for autonomous steering was carried out in December 2020 on “Głębokie” Lake in Szczecin (Figure 6).

**Figure 7:**
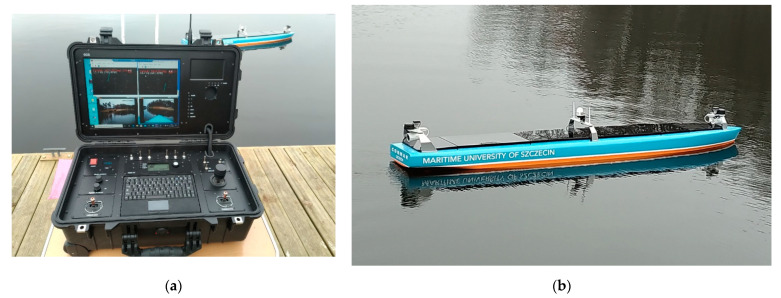
Experimental model of an unmanned, autonomous transport vessel with navigation equipment—tests on open water (“Głębokie” Lake): (**a**) land-based control station GCS; (**b**) unmanned ship model with computer system and navigation equipment.

During testing of the computer system and measurement and navigation equipment of the unmanned transport vessel, the model was controlled remotely and wirelessly from GCS. Figure 7 shows a commissioning GCS, which displays an image from two cameras and lidars and a model of the ship during the tests.

In order to achieve the assumed objectives of the experimental research, many tests and performance tests of the computer system and the measurement and navigation sensors installed in the model of unmanned ship were performed. One of the tests with the registration of all parameters is shown on the drawings and pictures below.

**Figure 8:**
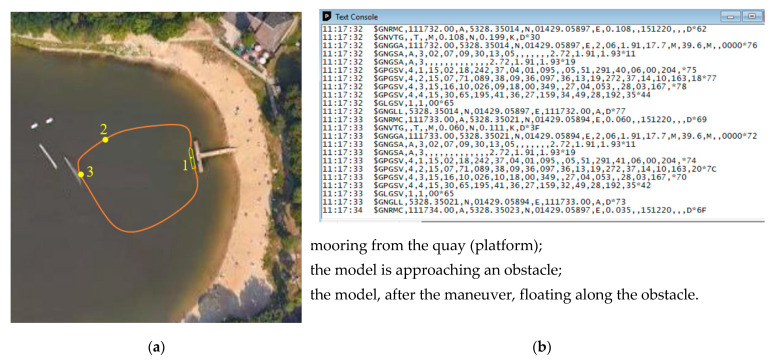
Registered (GNSS antennas and electronic compass) example of the movement trajectory of an unmanned ship model during one of the tests (the ship model is not drawn on the appropriate scale in relation to the quay (platform): (**a**) the movement trajectory of the ship model from one of the tests; (**b**) parameters recorded and saved from the GNSS antenna.

An example of the trajectory of the unmanned vessel model’s movement, drawn on a map of the basin on the basis of measuring the model’s position from the GNSS antenna, is shown in Figure 8.

**Figure 9:**
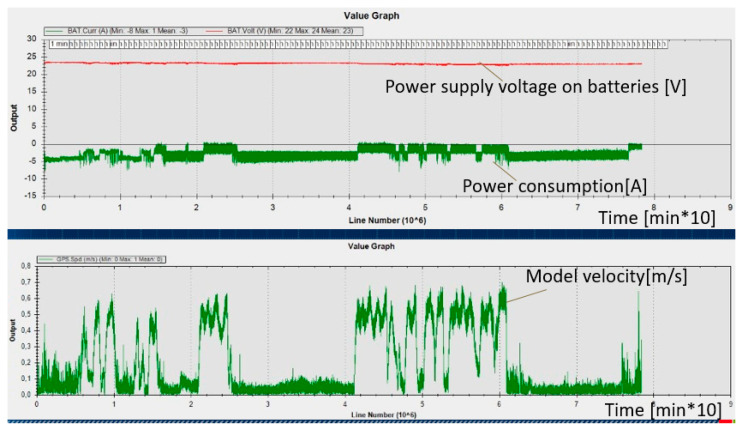
Example recording variables parameters of the ship model: speed (from a GNSS antenna) and power supply of one of the azimuthal thruster motors (from power measurement sensors).

In the ship model, during the tests, the parameters of the propulsion system were also measured in real time (model speed, power (voltage and current) of individual propulsion engines, battery capacity). An example record of the measured parameters is shown in Figure 9.

**Figure 10:**
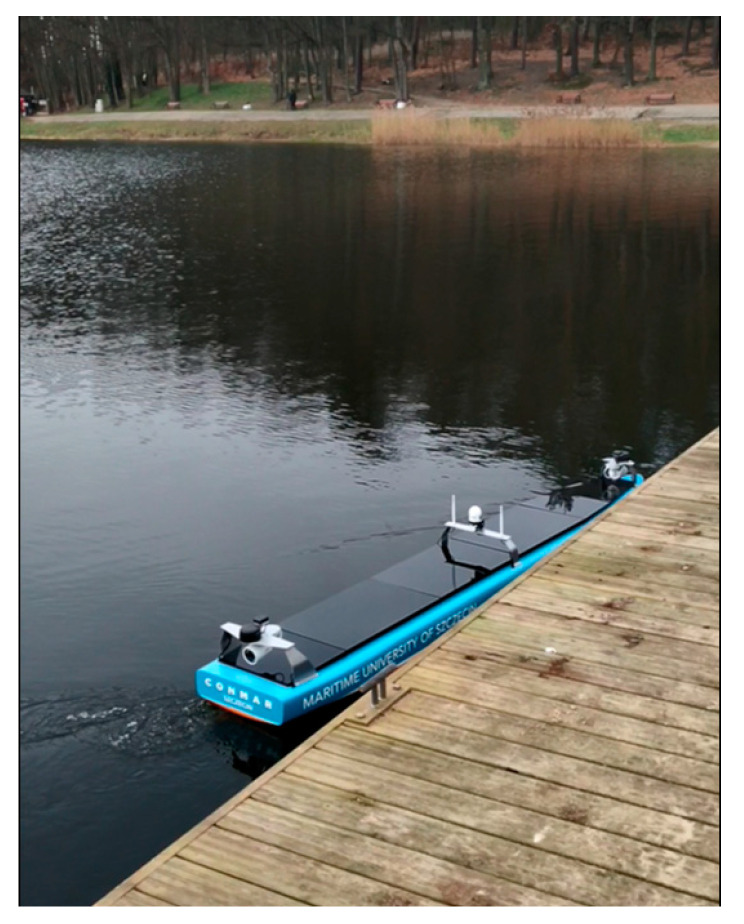
Unmooring maneuver from the platform (Pos. 1 from Figure 8)—photo of the model during unmooring from the platform.

**Figure 11:**
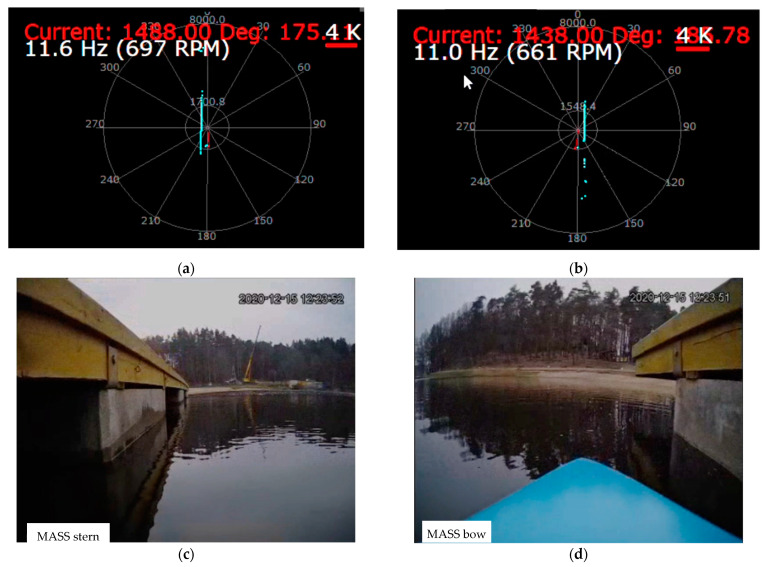
Unmooring maneuver from the platform (Pos. 1 from Figure 8)—snapshot from lidar and camera screens: (**a**) stern lidar image; (**b**) bow lidar image; (**c**) stern camera image; (**d**) bow camera image.

**Figure 12:**
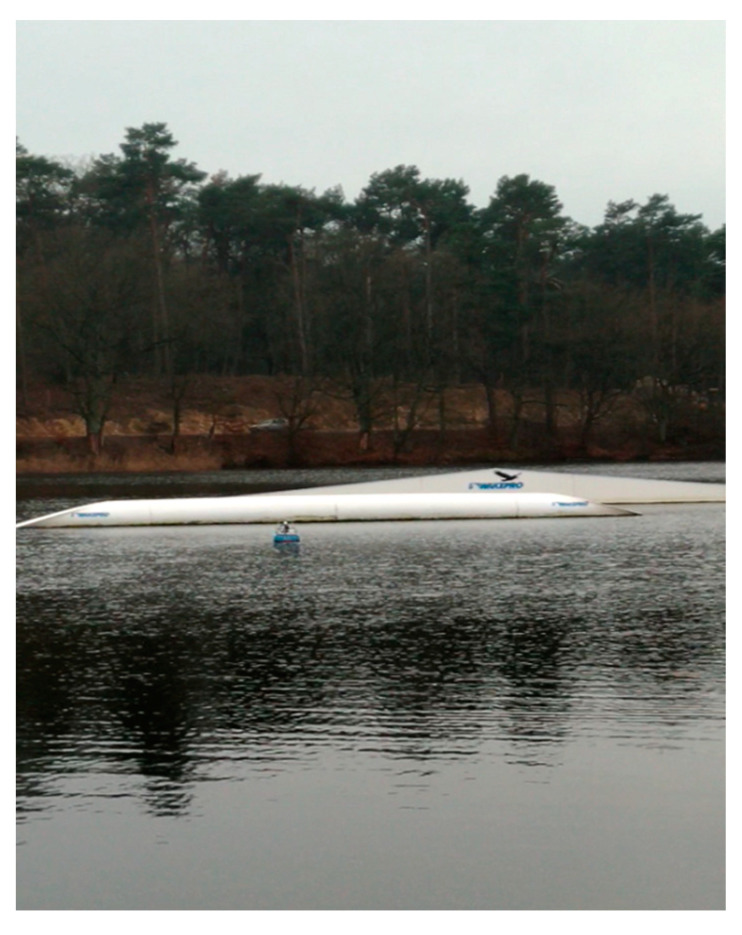
Model floating towards the obstacle (Pos. 2 from Figure 8)—photo of model floating towards the obstacle.

**Figure 13:**
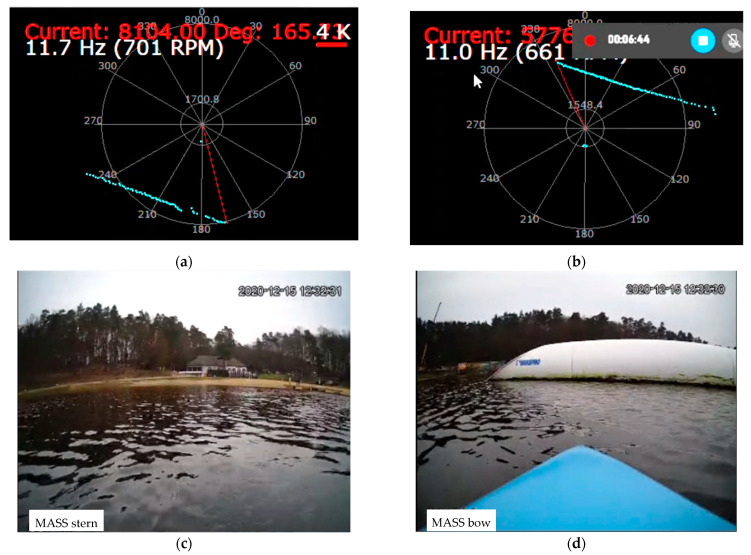
The model is floating towards the obstacle (Pos. 2 from Figure 8)—snapshot from lidar and camera screens: (**a**) stern lidar image; (**b**) bow lidar image; (**c**) stern camera image; (**d**) bow camera image.

**Figure 14:**
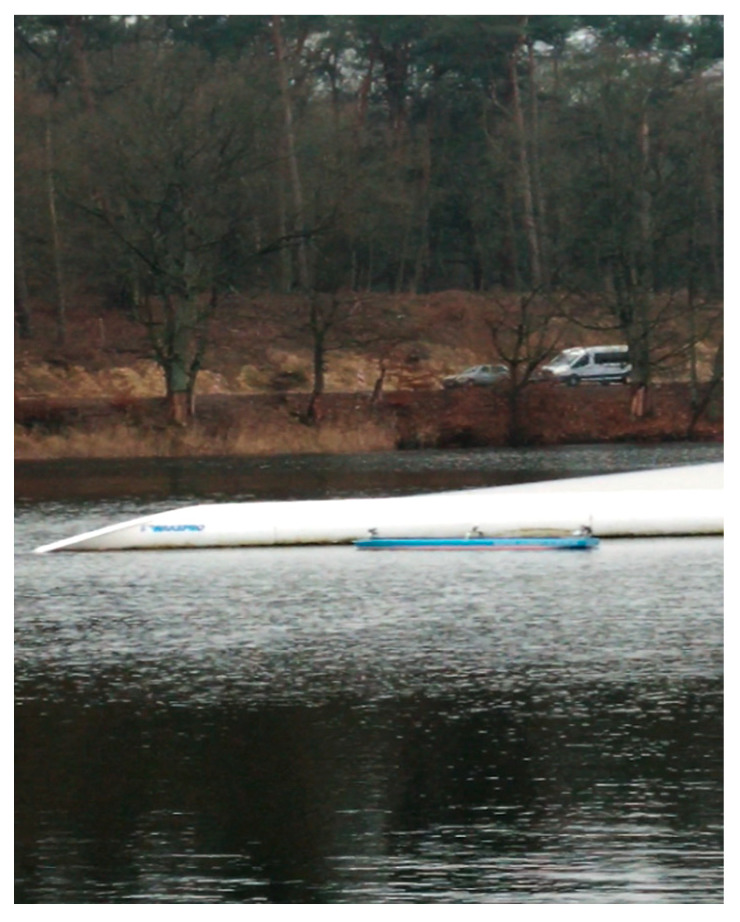
The model floating along a fixed obstacle (Pos. 3 from Figure 8)—a photo of the model floating along the obstacle.

**Figure 15:**
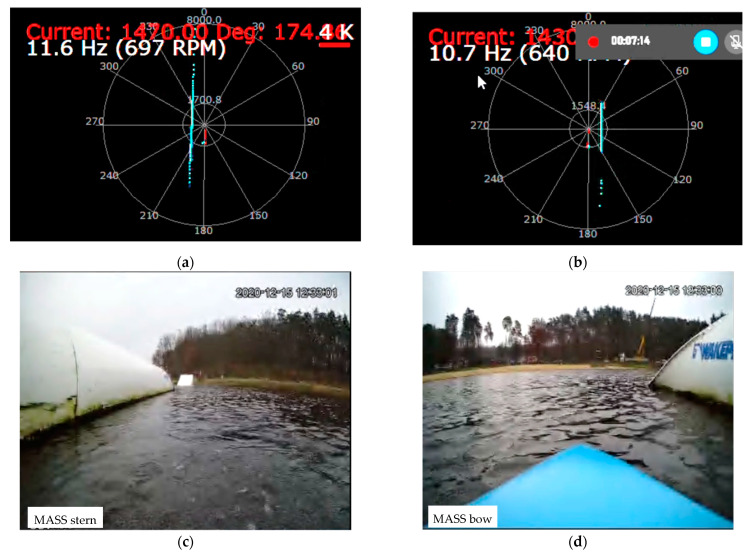
The model floating along a fixed obstacle (Pos. 1 from Figure 8)—snapshot from lidar and camera screens: (**a**) stern lidar image; (**b**) bow lidar image; (**c**) stern camera image; (**d**) bow camera image.

During the tests, the correctness of the operation of the cameras and laser lidars was checked, as well as the processing of the received image in the computer system for model control of the ship, image recording, and sending it to the land station. Example images from the cameras and lidars recorded during the test for the trajectory points from Figure 8 and the corresponding ship model images are shown in Figure 10 and Figure 11 (point 1), Figure 12 and Figure 13 (point 2), and Figure 14 and Figure 15 (point 3).

## 7. Discussion of Test Results

The main objective during the conducted tests was to determine whether the designed and built computer control system including all measurement and navigation sensors will be able to perform all tasks (functions) during autonomous control and whether the applied computer system will have sufficient computing power.

During the tests, the time of realization of individual computer operations and the delays occurring in the system were determined:

- During the software hang of the I/O module, the software Watchdog was 500 ms (can be reduced if necessary).
- The time delay for the I/O module to respond to a command from the PC is between 1.2 and 1.6 ms. To speed up communication, it is possible to change communication from ASCII to binary format. The system is equipped with a CAN bus, which can also be used for future data exchange between the PC and I/O module.
- The conversion time in the I/O module of the analog signals to digital was about 1 μs (12-bit converters). The total conversion time of analog values is, for 5 A/D channels, about 5 μs. Each measurement channel is software averaged and also filtered using a median filter for a sequence of 5 samples. The result is then converted to an output value (mA) and compared to a 5-point calibration curve for each sensor (calibration performed according to a certified current meter with adjustable current load).
- Approximate delay time between issuing the command from the PC to change the speed of the thruster and its execution is about 2–3 ms. The delay is the sum of communication time—UART, ASCII, decoding of information sent over the I2c bus (400 kHz) to the PWM generator, and the response of the generator itself.

The execution time of all operations (The execution time of all operations (processing, transmission, recording, computation)) was about 0.5 s, and the control loop delays were minimal, and, for the tested ship model, did not significantly affect its control. The model floats at a maximum speed of 1.64 m/s (Table 1), which is a very low speed relative to fast flying drones, for example, and during maneuvers, the model speed can be much lower.

The following conclusions can be drawn from the experimental study:

- All measuring sensors and navigational equipment were working properly and all recorded parameters were transmitted in real time to the control computer installed in the model vessel.
- Lidars were installed to detect obstacles on the water, regardless of the course of the ship in relation to the obstacles (the distance and position of the model ship in relation to the obstacle was determined on the basis of the image from the lidar).
- All recorded parameters from measuring sensors and navigation devices were properly registered in the on-board computer and prepared for transmission to the land station (GCS).
- All computational operations connected with recording and processing parameters from measurement sensors and navigation devices required from 10 to 15% of the total computational power of the on-board computer.
- After changing the broadband network frequency, the interference in the transmission of video from cameras and lidars no longer occurred; after a series of tests, it was concluded that the broadband transmission should be modified and the 5G LTE network should be used.

Based on the experimental study, it is concluded that the designed and built computer control system works properly and is optimal for the built unmanned autonomous vehicle model, and the on-board computer not only performs all computational processes, but still has reserves of computing power necessary for the expansion of the control system.

## 8. Conclusions

The presented computer system with a complete set of measurement and navigation devices, including the propulsion system of the ship model and the conducted experimental tests, can be considered as a new approach to research on autonomous control systems for unmanned ships. During the conducted tests, it was found that the technical parameters of the computer system were correctly selected for this unmanned ship model and the planned further research, which can also be considered as a new approach (such information is missing in the available literature). During the experimental tests, the computer hardware and the measurement and navigation equipment worked properly, although we anticipate some modifications (broadband transmission), and the results of the tests can be used for the design and construction of the computer control system of the actual unmanned vessel.

The information gathered from the tests and the conclusions drawn allow the development of guidelines for the design of a computer system for a real ship that will travel at a much higher speed (Table 1) than the tested ship model.

Since the hardware tests so far are positive, the next stage of the research will be to test the software for autonomous control of the ship model. 

The control software consists of many modules—the autonomous ship must independently implement various maneuvers resulting from different situations arising during the voyage from the starting port to the destination port.

## Figures and Tables

**Table 1:** Technical and operational parameters of an unmanned vessel.

Parameter	Unit	Ship	Model (Scale 1:25)
Length over all L_oa_	m	78.75	3.15
Length between perpendiculars L_PP_	m	75.00	3.00
Breadth B	m	11.10	0.47
Draft T	m	4.33	0.17
Displaced value ∇	m^3^	2500.00	0.16
Block coefficient c_B_	-	0.6589	0.6589
Velocity V	m/s	8.22	1.64

## Data Availability

Not applicable. (All results and data are included in the manuscript.)
